# Atypical Response Regulator ChxR from *Chlamydia trachomatis* Is Structurally Poised for DNA Binding

**DOI:** 10.1371/journal.pone.0091760

**Published:** 2014-03-19

**Authors:** Michael L. Barta, John M. Hickey, Asokan Anbanandam, Kevin Dyer, Michal Hammel, P. Scott Hefty

**Affiliations:** 1 Department of Molecular Biosciences, University of Kansas, Lawrence, Kansas, United States of America; 2 Department of Pharmaceutical Chemistry, University of Kansas, Lawrence, Kansas, United States of America; 3 Del Shankel Structural Biology Center, University of Kansas, Lawrence, Kansas, United States of America; 4 Physical Biosciences Division, Lawrence Berkeley National Laboratory, Berkeley, California, United States of America; Oregon State University, United States of America

## Abstract

ChxR is an atypical two-component signal transduction response regulator (RR) of the OmpR/PhoB subfamily encoded by the obligate intracellular bacterial pathogen *Chlamydia trachomatis*. Despite structural homology within both receiver and effector domains to prototypical subfamily members, ChxR does not require phosphorylation for dimer formation, DNA binding or transcriptional activation. Thus, we hypothesized that ChxR is in a conformation optimal for DNA binding with limited interdomain interactions. To address this hypothesis, the NMR solution structure of the ChxR effector domain was determined and used in combination with the previously reported ChxR receiver domain structure to generate a full-length dimer model based upon SAXS analysis. Small-angle scattering of ChxR supported a dimer with minimal interdomain interactions and effector domains in a conformation that appears to require only subtle reorientation for optimal major/minor groove DNA interactions. SAXS modeling also supported that the effector domains were in a head-to-tail conformation, consistent with ChxR recognizing tandem DNA repeats. The effector domain structure was leveraged to identify key residues that were critical for maintaining protein - nucleic acid interactions. In combination with prior analysis of the essential location of specific nucleotides for ChxR recognition of DNA, a model of the full-length ChxR dimer bound to its cognate *cis*-acting element was generated.

## Introduction

Two-component signal transduction systems (TCS) are a fundamental mechanism employed by bacteria for rapid adaptation to environmental changes. TCS typically consist of a membrane-bound sensor histidine kinase (HK) and an associated response regulator (RR). Upon sensing stimuli, the sensor kinase undergoes an autophosphorylation event from which the phosphoryl group is then transferred to the receiver domain of a cognate RR. Phosphorylation of the RR promotes oligomerization, stabilizing the active form of the protein. The majority of response regulators contain a DNA-binding domain that alters gene expression in response to phosphorylation [1]. The functions of RRs involve a diverse array of responses, including drug resistance, motility, osmoregulation, pathogenic host invasion and phosphate uptake, among others [2]. RRs are subdivided into families based upon the structure/function of their DNA binding domains. The largest subfamily of RRs (OmpR/PhoB) is comprised of a winged helix-turn-helix domain [3].

Members of the OmpR/PhoB RR subfamily are composed of a receiver domain that contains the site of phosphorylation and homodimerization, and an effector domain that interacts with DNA through the subfamily-defining winged helix-turn-helix motif and RNA polymerase machinery through a transactivation loop [4]. Effector domains of OmpR/PhoB subfamily members share a common tertiary structure, which results in a conserved DNA binding mechanism. The typical OmpR/PhoB effector domain is comprised of an N-terminal four strand β-sheet, a helix-turn-helix motif and a C-terminal β-hairpin wing [4]. DNA interaction is achieved primarily through electrostatic interactions between residues within the helix-turn-helix motif and the DNA major groove. DNA binding is further stabilized through interactions between residues within the wing of the effector domain and the adjacent DNA minor groove. While the overall topology of effector domains is conserved, the distinct functional characteristics (e.g. specific DNA binding residues) associated with individual OmpR/PhoB effector domains are predominately provided by differences in key residues and side chain orientation.

OmpR/PhoB interdomain interactions and overall conformations are diverse and appear to reflect the relative DNA ‘on-off’ equilibrium for an individual RR [5]. For instance, full-length protein structures and functional studies of MtrA [6] and PrrA [7] from *M. tuberculosis* and DrrB [8] from *T. maritima* support that the receiver domain forms an extensive intramolecular interface with the effector domain effectively occluding the α4-β5-α5 dimerization interface and resulting in an equilibrium skewed towards an inactive (off) state [5]. In contrast, RegX3 [9] and PhoP [10] from *M. tuberculosis* and DrrD [11] from *T. maritima*, have relatively limited interdomain interfaces for which the DNA binding regions are in solvent accessible positions. These structures reflect the ability of unphosphorylated forms to bind DNA, albeit at lower affinity than phosphorylated forms, in a weak on-state that is enhanced by phosphorylation and stabilized by homodimerization. Importantly, these observations are from a collection of full-length structures of *unphosphorylated* OmpR/PhoB response regulators limiting our understanding of the structural and functional diversity employed by this large subfamily.

A relatively new subset of response regulators are the atypical RRs which do not require phosphorylation for activity and have been described in a broad range of phylogenetically diverse organisms. The receiver domain active site typically lacks conserved residues involved in phosphorylation, yet maintains structural homology with prototypical OmpR/PhoB RRs [12], [13]. Recent reports have revealed that atypical RRs can exist as monomers [14] or dimers [12] and exhibit a relatively strong affinity for target DNA in the absence of phosphorylation. It remains unclear which structural aspects allow atypical RRs to function in a phosphorylation-independent state, however it is likely these mechanisms retain a large degree of similarity to canonical OmpR/PhoB subfamily members. Of note is HP1043 from *H. pylori* for which a full-length NMR solution structure has been determined [12]. This solitary atypical OmpR/PhoB structure revealed that the effector domain is in a distinct, free-open state with virtually no interactions with the receiver domain. These structural and functional observations support that this atypical response regulator is predominantly in an ‘on state’ in the absence of phosphorylation.

ChxR is an atypical OmpR/PhoB subfamily response regulator encoded by the medically important bacterial pathogen *Chlamydia* [13], [15], [16]. Similar to HP1043, ChxR lacks several conserved active site residues, including the phospho-accepting Asp and is able to activate transcription in a phosphorylation independent manner, leading to its classification as an atypical RR [13], [15]. Additionally, ChxR exists as a stable homodimer in the absence of phosphorylation, while recognizing multiple sites within its own promoter [13]. ChxR shares 30% identity across the entire HP1043 polypeptide and only 22% identity within the effector domain. Importantly, a contrasting feature between ChxR and HP1043 is the binding of direct or inverted repeats, respectively, indicating a difference in DNA binding domain orientation. Previous studies have suggested that ChxR has a more global role in *Chlamydia* gene expression based upon the relatively high number of potential binding sites [17]. Additionally, ChxR expression analysis supports that it likely exerts its biological role during developmental stages that include infectious *Chlamydia* formation [*17*]. Despite previous studies that have characterized numerous characteristics of ChxR, including DNA recognition sequences and motif [13], [15], the residues and regions critical for DNA binding have not been identified. Solution structure studies on both the effector domain and full-length ChxR were carried out, in order to better understand how ChxR interacts with cognate DNA. These observations guided additional functional analyses and the development of a proposed model of full-length ChxR interacting with its cognate direct repeat DNA.

## Materials and Methods

### Protein Purification

The effector domain of ChxR (ChxR_Eff_) and full-length ChxR (ChxR) were purified as previously described [17], [18]. Briefly, each protein was expressed in *E. coli* BL21(DE3) (Invitrogen, Carlsbad, CA) and initially purified through metal (Co^2+^) affinity chromatography. Following their elution from the affinity column, each protein was further purified by size exclusion chromatography and determined to be >95% pure by Coomassie staining after SDS-PAGE.

### NMR Spectroscopy

ChxR_Eff_ was overexpressed in *E. coli* BL21(DE3) cells and ^13^C/^15^N labeled using a previously established method [19]. Following expression, ChxR_Eff_ was purified as described above. The purified protein was equilibrated in 20 mM Na_2_HPO_4_ pH 6.5, 20 mM KH_2_PO_4_, 20 mM NaCl, and 1 mM DTT and then concentrated to 1.5 mM using an Amicon (Millipore, Billerica, MA) 3,000 molecular weight cut-off centrifugal device.

The sample for NMR spectroscopy experiments was comprised of 90% 1.5 mM ChxR_Eff_ and 10% D_2_O. All NMR spectra were recorded on a BRUKER AVANCE 800 MHz NMR instrument equipped with a TCI cryoprobe. All NMR experiments were recorded at 25°C. Sequential assignments of the backbone resonances of ChxR_Eff_ were achieved by 2D and 3D- hetero nuclear triple resonance NMR experiments, 2D-^1^H-^15^N-HSQC, 2D-^1^H-^13^C-HSQC, 3D-HNCA, 3D-HNCO, 3D-HNCACB, 3D-CBCA(CO)NH, 3D-HBHA(CBCACO)NH, 3D-HBHANH. Side chain assignments were obtained from 3D-H(CCCO)NH, and 3D-HCCH-TOCSY experiments [20]. All NMR spectra were processed using *NMRPipe* [21] and analyzed with *SPARKY* [22].

For the ChxR_Rec_-ChxR_Eff_ chemical shift titration experiment, ChxR_Eff_ was ^15^N labeled and purified as described herein and ChxR_Rec_ was expressed and purified as described previously [13]. After an initial 2D-^1^H-^15^N-HSQC was taken of 1 mM ChxR_Eff_, unlabeled ChxR_Rec_ was titrated into the protein sample. 2D-^1^H-^15^N-HSQCs were taken at 2∶1 and 1∶1 molar ratios of ChxR_Eff_ to ChxR_Rec_.

### NMR Structure Calculation

Structures were calculated by restrained simulated annealing using NOE based distance restraints and *TALOS+* [23] based dihedral angle restraints. The torsion angle dynamics protocols of *CNS* 1.2 [24] were used to calculate 50 structures that were then refined using Cartesian dynamics. The 25 structures with the lowest total energies were selected for subsequent analysis. None of the 25 structures violated any distance restraints more than 0.5 Å or any dihedral angle restraints more than 5.0°. Structures were analyzed using *PROCHECK-NMR* [25]. Approximate interproton distances were obtained from ^15^N and ^13^C edited NOESY-HSQC experiments. The mixing time was 100 ms for ^15^N-edited NOESY and 120 ms for ^13^C-edited NOESY NMR experiments. The distance restraints were subdivided into four groups on the basis of NOE peak intensities: 1.8–2.8 Å for strong NOEs, 1.8–3.4 Å for medium NOEs, 1.8–5.0 Å for weak NOEs and 1.8–6.0 Å for very weak NOEs. In addition, backbone dihedral angles φ, and ψ determined using *TALOS+* were restrained to −60°±35° (φ) and −40°±30°(ψ) for α-helical regions. For β-strands the values were taken as φ = −120°±30° and ψ = 120°±30°. Final statistics are listed in Table 1.

**Table 1:** Structural statistics of the 25 lowest energy NMR structures of ChxR_Eff_.

**Distance Restraints**	
Total NOE	1637
Intraresidue (|i−j| = 0)	130
Sequential (|i−j| = 1)	531
Medium-range (1<|i−j|≤5)	406
Long-range (|i−j|≥5)	570
**Dihedral restraints**	
φ (*TALOS+*)^a^	94
ψ (*TALOS+*)	94
**R.m.s. deviations from experimental restraints** a	
NOE-based distance restraints (Å)	0.016±0.0008
Dihedral angle restraints (°)	0.209±0.0270
**R.m.s. deviations from idealized geometry**	
Bonds (Å)	0.0020±0.0000
Angles (°)	0.3495±0.0099
Impropers (°)	0.2166±0.0131
**R.m.s. deviations from the mean structure (Å)** b	
Backbone atoms (N, C^α^, C’)	0.76±0.14
All Heavy Atoms	1.62±0.13
**Ramachandran plot** c	
Residues in most favored regions, %	68.3
Residues in additional allowed regions, %	29.1
Residues in generously allowed regions, %	2.5
Residues in disallowed regions, %	0.1
**PDB ID**	2M1B

a These values are for 25 lowest energy structures out of 50 structures.

b Only secondary structural elements are superimposed.

c For the 25 lowest energy structures, using *PROCHECK-NMR.*

### NMR Relaxation Studies

^15^N T1 and T2 relaxation times and values for the ^1^H-^15^N NOE were measured on a 600 MHz VARIAN INOVA Spectrometer using standard pulse sequences [26]. Delays of 10, 60, 130, 230, 360, 520, 720, 960 and 1.5 ms for T1 and 10, 30, 50, 70, 90, 110, 130, 150, 170 and 190 ms for T2 values were used. Values for T1 and T2 were determined by plotting the peak heights versus delay times and fitting the curve to a mono exponential nonlinear least squares fit, available in NMR data analysis software *SPARKY* [22]. The rotational correlation time (τ_C_; ns time scale) of a monomeric protein (<25 kDa) in solution is approximately 0.6 times its molecular weight (kDa). The τ_C_ value was calculated from the following equation [27]:
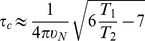



### Electrophoretic Mobility Shift Assay

Electrophoretic mobility shift assays were performed as described for ChxR with DNA corresponding to the high-affinity (DR2) binding site within the *chxR* promoter [17]. The binding reactions contained 1 nM DNA and 50 nM, 100 nM, 500 nM, 1 μM, 5 μM or 10 μM ChxR_Eff_. The assay was performed in triplicate and the amount of DNA shifted was visualized and quantified using the Odyssey Infrared Imaging System (LI-COR Biosciences, Lincoln, NE).

*Site-directed Mutagenesis* - Mutations were introduced into the full-length ChxR plasmid [17], [18] using the QuikChange II XL site-directed mutagenesis kit by following the manufacturer’s protocol (Agilent Technologies, La Jolla, CA). All clones were verified by DNA sequencing analysis (ACGT, Inc., Wheeling, IL).

### SAXS Data Collection and Evaluation

SAXS data were collected at the ALS beamline 12.3.1 (SIBYLS) LBNL Berkeley, California [28]. Data were collected using a wavelength λ = 1.0 Å and with the sample-to-detector distance set to 1.5 m resulting in scattering vectors, q, ranging from 0.01 Å^−1^ to 0.33 Å^−1^. The scattering vector is defined as q = 4π sinθ/λ, where 2θ is the scattering angle. All experiments were performed at 20°C and data was processed as previously described [28].

SAXS data at short and long time exposures (0.5, 1 and 4 s) were merged to define the entire scattering profile. Different protein concentrations were tested for aggregation and examined by Guinier plots. The radius of gyration (R_g_) was derived by the Guinier approximation I(q) = I(0)*exp(-q^2^R_g_^2^/3) with the limits qR_g_ <1.3. The curves measured for different protein concentrations (1.25, 2.5, 5.0 mg/ml) displayed a concentration dependence arising from inter-particle interaction (attractions) at q <0.05 Å^−1^ and interference free scattering profiles were estimated by extrapolating the measured scattering curves to infinity dilution (see **Fig. S4A** and [29]). The program SCATTER was used to compute the pair-distance distribution functions, P(r) and to perform Porod–Debye analysis to obtain the P coefficient and Porod Volume [30], which indicated a dimeric state of ChxR with estimated MW = ∼62 kDa (calculated MW = ∼56 kDa).

The overall shape was restored from the experimental data using the program DAMMIF with P_1_ symmetry operator [31]. In our rigid body modeling strategy BILBOMD, molecular dynamics (MD) simulations were used to explore conformational space adopted by the ChxR C-terminal effector domain, which we connected to the N-terminal receiver domain via an 8 residue-long flexible linker. For each registered conformation, the theoretical SAXS profile and the corresponding fit to the experimental data were calculated using the program FoXS [32]. Two sets of ChxR models were generated, one with zero constraints on the orientation of the two effector domains (*unconstrained*) and one requiring that a head-to-tail orientation of the effector domains be maintained (*constrained*). The *unconstrained* model set allowed for all possible orientations of the linker and effector domain, relative to the receiver domain dimer. All possible orientations were also generated for the *constrained* model set, with the requirement that a head-to-tail orientation be maintained for the effector domains. A Minimal Ensemble Search (MES) was ultimately used to select two conformers from a pool of all generated *constrained* conformers that achieved the best fit (χ) to the experimental curve [33]. Chi is defined by the following equation:
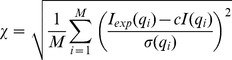



Comparison of the structural properties of the selected conformers allowed us to distinguish the degree of flexibility and heterogeneity of the experimental system [34]. Additional SAXS parameters are available in **Table 2**.

**Table 2:** SAXS Parameters for Data Validation and Interpretation.

SAXS parameters		Comments
q-range (Å^−1^)	0.01–0.33	
I(0)	8e^2^±5.4e^0^	Intensity at q = 0
R_g_ (Å)	30.07±0.89	R_g_ value was obtained after extrapolationto infinite dilution; single values werecalculated from Guinier fit using a q*R_g_ <1.6
R_g_ (Å) real	31.53±0.08	R_g_ values were calculated from the P(r)function by the program SCATTER [30].
V (Å^3^)	1.47e^5^	Volume was determined based on [54]
Mw ^SAXS^	6.14e^4^±0e^0^	Mw was estimated from the Volumebased on [54]
D_max_ (Å)	100	Maximal dimension was determined fromP(r) function
P	3.8	Porod Exponent
**Structure Modeling**		
Quality of Fit (χ) -singlebest model	3.86	Scattering profiles up to q_max_. 0.33 Å^−1^ andfit for the atomic models were calculated bythe program FoXS [32]
Quality of Fit (χ) -MES	3.66	Minimal Ensemble fit was obtained by MES [33]
Quality of Fit (χ) -singlebest unconstrained model	8.05	Scattering profiles up to q_max_. 0.33 Å^−1^ andfit for the unconstrained atomic models werecalculated by the program FoXS [32]
SAXS shape (NSD)	0.79±0.07	The values given are the average and standarderror from ten runs of the DAMMIF [31]

### Multiple Sequence Alignments and Figure Modeling

Multiple sequence alignments were carried out using ClustalW [35] and aligned with secondary structure elements using ESPRIPT [36]. OmpR/PhoB RR effector domain sequences used in alignments, along with their respective GenBank accession numbers, were as follows: ChxR, *C. trachomatis*, 15605361; PhoB, *E. coli*, 213521171; OmpR, *E. coli*, 242378928; HP1043, *H. pylori*, 15645657; DrrB, *T. maritima*, 15642901; RegX3, *M. tuberculosis*, 15607632; MtrA, *M. tuberculosis*, 509815; PrrA, *M. tuberculosis*, 397672721; DrrD, *T. maritima,* 15643165. Three-dimensional structures were superimposed using the Local-Global Alignment method (LGA) [37]. OmpR/PhoB RR structures were obtained from the PDB [38] and are as follows: PhoB (1QQI); YycF (2D1V); HP1043 (2HQR); OmpR (2JPB); PhoP (2PMU); KdpE (3ZQ7) for effector domains and DrrD (1KGS); DrrB (1P2F); PrrA (1YS6); MtrA (2GWR); HP1043 (2HQR); RegX3 (2OQR); PhoP (3R0J) for full-length structures. Representations of all structures were generated using PyMol [39]. Calculations of electrostatic potentials at the molecular surface were carried out using DELPHI [40]. All figure representations of full-length ChxR have the N-terminal fusion tag and disordered C-terminal (truncated after final secondary structure element) region removed for clarity. Numbering of all residues in this work reflects their position in the *C. trachomatis* ChxR sequence. Secondary structure elements are numbered with respect to their position in full-length ChxR.

### Accession Numbers

The atomic coordinates and structure factors (code 2M1B) have been deposited in the Protein Data Bank, Research Collaboratory for Structural Bioinformatics, Rutgers University, New Brunswick, NJ (http://www.rcsb.org/) as well as Biological Magnetic Resonance Data Bank (http://www.bmrb.wisc.edu/) (code 17014).

## Results

### ChxR_Eff_ alone can Bind DNA

We have previously shown that ChxR interacts with DNA corresponding to the DR2 (5′-TCGATCA-N_5_-TAGATAA-3′) binding site in the *chxR* promoter with a dissociation constant (K_d_) of approximately 44 nM [17]. To determine if ChxR_Eff_ (residues 115–227) alone can to bind to DNA, an electrophoretic mobility shift assay (EMSA) was performed with the DR2 DNA sequence. Indeed, ChxR_Eff_ can bind to DNA (**Fig. S1**), albeit at a much weaker affinity than full-length ChxR. Increasing concentrations of ChxR_Eff_ (50 nM–10 μM) were quantified with respect to DNA interaction and a K_d_ was calculated (450±75 nM). The calculated K_d_ assumes that two ChxR_Eff_ molecules are bound to the DNA as the DR2 sequence contains two binding sites. The approximate 10-fold decrease in DNA affinity for ChxR_Eff_ relative to ChxR likely results from the lack of receiver domain-mediated dimerization to stabilize the protein-nucleic acid complex. This result is in agreement with previous studies on OmpR/PhoB RRs that demonstrated dimerization promotes DNA interaction [13].

### Structural Analysis of ChxR_Eff_

We have previously elucidated the structure of the ChxR receiver domain (ChxR_Rec_), which has many unique features compared to typical OmpR/PhoB subfamily members [13]. To determine if the atypical features of ChxR are limited to the receiver domain or if the effector domain also has unique features, we determined the solution structure of the effector domain (ChxR_Eff_) through NMR Spectroscopy. Analysis of the structure of ChxR_Eff_ will help facilitate the identification of residues important to DNA binding.

**Figure 1:**
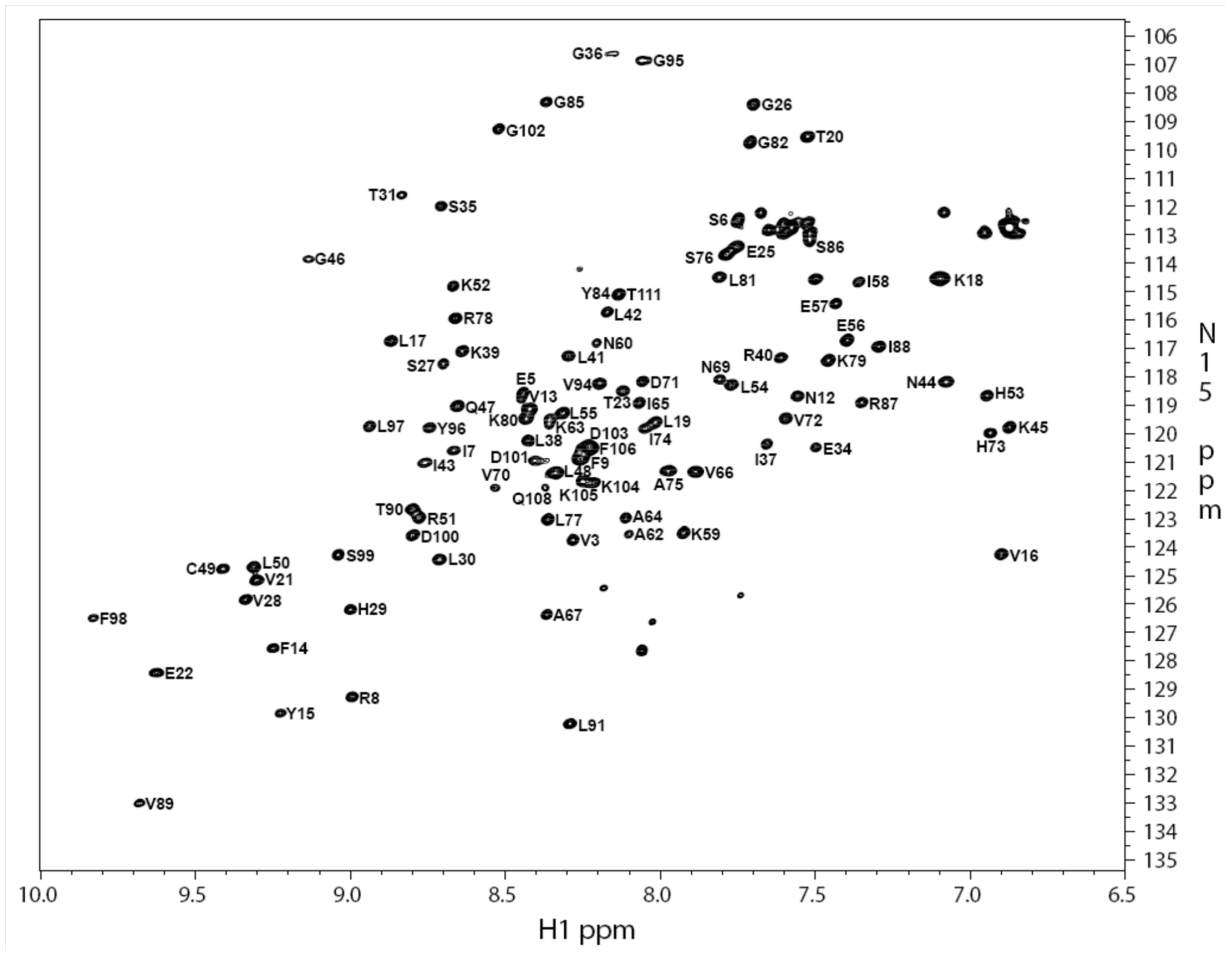
^1^H-^15^N HSQC spectrum of ChxR_Eff_. 2D ^1^H-^15^N HSQC spectrum of 1.5 mM ^15^N, ^13^C labeled ChxR_Eff_ acquired on a BRUKER AVANCE 800 NMR spectrometer at 25°C. Cross peaks are labeled with their corresponding backbone assignments.

To determine whether an NMR approach was suitable for investigating the structure of ChxR_Eff_, an initial Heteronuclear Single Quantum Coherence (HSQC) spectrum was analyzed with a ^1^H-^15^N-labeled sample of ChxR_Eff_. Resonance signals for 105/112 residues were detected (**Fig. 1**). The signals were well resolved and dispersed, which was a positive indication that the structure of the protein could be determined using this method. Following data acquisition and analysis as described in the *Materials and Methods* section, the 25 lowest total energy structures (**Fig. S2**) displaying good Ramachandran plot statistics and low restraint violations were selected for further analysis. An average of 15 NOEs per residue (1637/112) constrains the ChxR_Eff_ structure, while 84% (94/112) of the dihedral angles were defined. The RMSD of the backbone atoms of the mean structure was 0.76±0.14 Å, indicating a high degree of structural similarity across the 25 lowest energy structures. All relevant NMR statistics are listed in **Table 1** and the final structure of ChxR_Eff_ was deposited in the Protein Data Bank under the identification number 2M1B and the Biological Magnetic Resonance Data Bank under code 17014.

**Figure 2:**
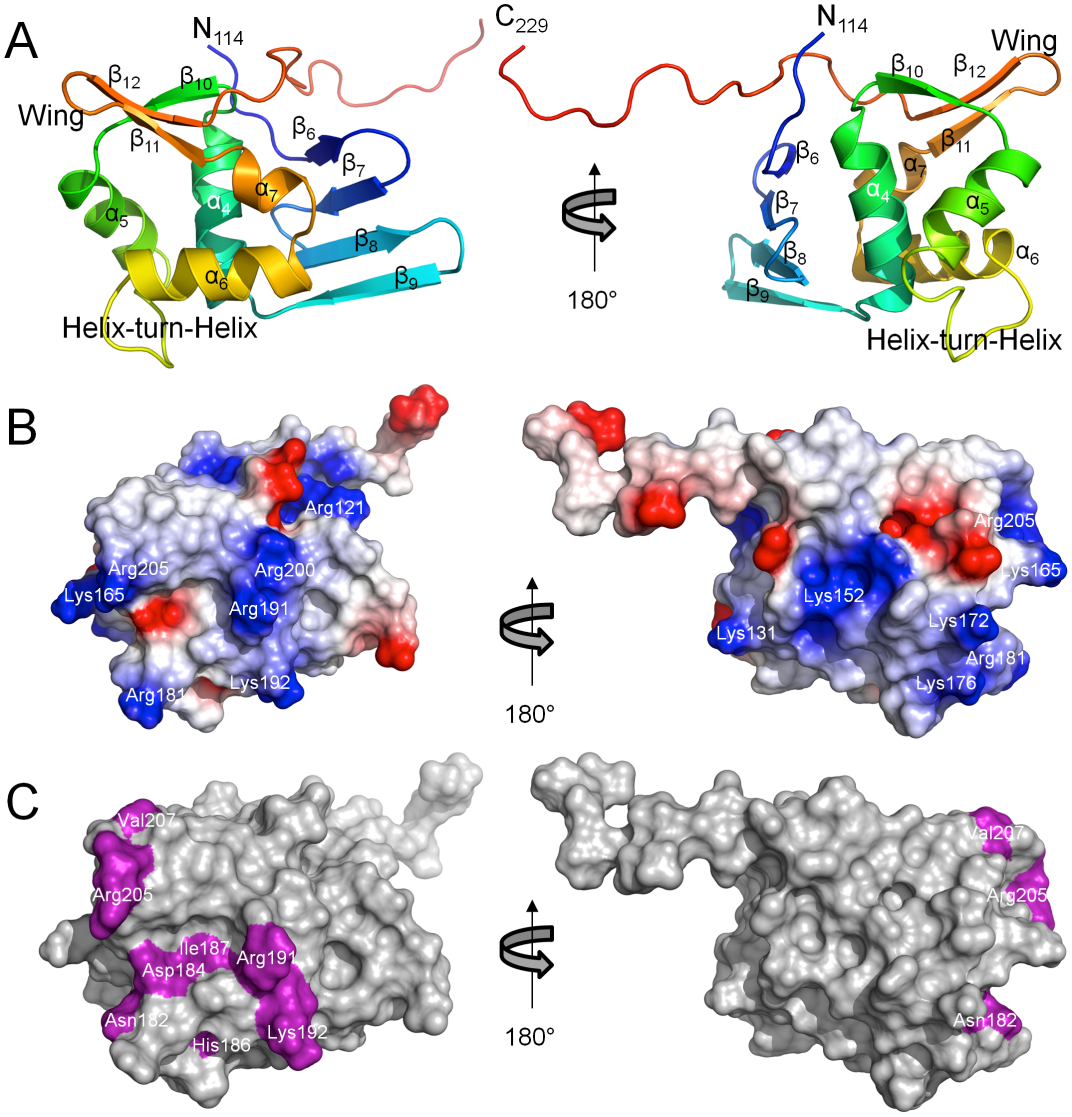
Solution structure of ChxR_Eff_. *A,* Cartoon ribbon diagram of the minimized mean structure of *C. trachomatis* ChxR_Eff_ (residues 114–229) colored blue (N-terminus) to red (C-terminus). The topology of ChxR_Eff_ is β6–β7–β8–β9–α4–β10–α5–α6–α7–β11–β12. *B,* Surface representation of electrostatic potential, generated by DelPhi [40], of ChxR_Eff_. Color scheme represents regions of negative (*red*) and positive (*blue*) charge density contoured at ±3 *e*/*kT*. Structure is oriented the same as panel A. *C,* Surface representation of ChxR_Eff_ with surface exposed side chains targeted for site directed mutagenesis colored magenta. Structure is oriented the same as panel A. All three panels are rotated 180° on the *right*.

The structure of ChxR_Eff_ (**Fig. 2A**) is comprised of a four-stranded antiparallel β-sheet (β6–β7–β8–β9, residues Ile120-Phe122, Asn125-Tyr128, Leu132-Thr136 and Gly139-Leu143, respectively), followed by one α-helix (α4, residues Pro145-Asn157), one β-sheet (β10, residues Gly159-Cys162), three α-helices (α5–α6–α7, residues Arg164-Asn173, Val183-Leu194 and Ala196-Arg200, respectively) and a β-hairpin (β11–β12, residues Ile201-Leu204 and Val207-Phe211, respectively)^#^. Lastly, the C-terminus of ChxR_Eff_ is a long random coil (residues Ser212-Glu229), characterized by a high degree of conformational flexibility (**Fig. S2**). The overall topology of ChxR_Eff_ is β6–β7–β8–β9–α4–β10–α5–α6–α7–β11–β12.

The backbone dynamics of ChxR_Eff._ were investigated by ^15^N T1, T2 and Heteronuclear-NOE, with an average T1 value of 0.43±0.06 s and an average T2 value of 0.064±0.018 s. Het-NOEs for well-defined regions in the structure were found to be in the range of 0.75–0.85 indicating that internal motions on the ps (fast motion) time scale are restricted. Het-NOEs for the N- and C-termini as well as loop residues 174–182 were significantly smaller than average. The overall rotational correlation time (τ_C_) was estimated to be 8.66±0.40 ns from T1/T2 ratios of residues selected from well-defined regions of the structure. This value indicates that ChxR_Eff_ exists in a monomeric state in solution (MW in kDa×0.6; expected τ_C_ for monomeric ChxR_Eff_ (12.7 kDa) = 7.6 ns) and is in good agreement with those reported in the literature for proteins of similar size [26], [27]. In additional support of a monomeric state, additional and/or broad cross peaks were not observed in the 2D-HSQC spectrum.

**Figure 3:**
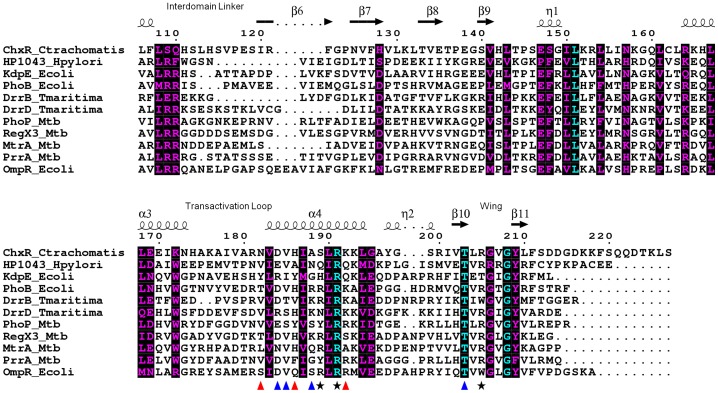
Limited structure-based sequence alignment of OmpR/PhoB subfamily Response Regulator effector domains. Numbers above the sequences correspond to *C. trachomatis* ChxR. The secondary structure of ChxR is shown above the alignment. Residues are colored according to conservation (cyan = identical and purple = similar) as judged by the BLOSUM62 matrix. Red triangles below the sequences correspond to amino acid side chains identified by ChxR mutagenesis that are involved in DNA binding, blue triangles correspond to DNA interacting side chains identified within a single OmpR/PhoB subfamily member while black stars represent DNA interaction sites within multiple OmpR/PhoB subfamily members [41], [43]–[45]. Sequences used within alignment are comprised of OmpR/PhoB subfamily members with extensive structural and/or functional studies. Accession numbers are detailed in the *Materials and Methods* section.

The electrostatic surface potential of ChxR_Eff_ (**Fig. 2B**) reveals several regions of positive charge on both faces of the protein. Of potential importance is a cohort of Arg (121, 181, 191, 200 and 205) and Lys (165 and 192) residues within the putative DNA binding region (helix-turn-helix and wing motifs). Positive surfaces appear to be conserved within OmpR/PhoB subfamily effector domains [*3*], [41], [42], as would be expected from a region that interacts with the negatively charged phosphate backbone of DNA. Unsurprisingly, several of these residues have previously been implicated in DNA binding studies [12], [41], [43]–[45] (Arg191, Lys192 and Arg205) and are highly conserved across this subfamily (**Fig. 3**).

### ChxR_Eff_ Resembles Prototypical OmpR/PhoB Subfamily Members

In contrast to the atypical receiver domain dimerization interface, the structure of ChxR_Eff_ closely resembles that of typical OmpR/PhoB subfamily effector domain structures. While the primary sequence similarity between effector domains of the OmpR/PhoB subfamily varies from 20–65% [3], the secondary and tertiary structure of this domain is highly conserved throughout the subfamily. Structural superposition with previously determined OmpR/PhoB subfamily effector domains (OmpR [3], [43], [46], PhoB [42], PhoP [47], KdpE [45] and HP1043 [12]) reveals ChxR_Eff_ aligns with an RMSD no greater than 2.50 Å to each structure (**Table 3**, **Fig. S3**). The high structural similarity between OmpR/PhoB subfamily effector domains has been proposed to be a result of the conservation of 13 hydrophobic residues that comprise the hydrophobic core of the effector domain [48]. Indeed, twelve of the thirteen residues are conserved in ChxR_Eff_.

**Table 3:** Superposition Analysis of ChxR_Eff_ with OmpR/PhoB Subfamily Effector Domains.

OmpR/PhoB Effector Domain	Corresponding Cα Positions^a^	RMSD (Å)	Sequence Identity (%)	LGA_S^b^
OmpR	88/104	2.49	25.0	51.2
PhoB	83/104	2.36	28.9	53.6
PhoP	78/98	2.38	25.6	47.1
KdpE	86/101	2.37	25.6	49.0
HP1043	82/106	2.38	22.0	47.8

a Denotes the number of Effector Domain residues that superimpose within 5.0 Å distance of an equivalent position in ChxR_Eff._

b The LGA_S parameter represents a scoring function to evaluate the overall levels of structural similarity between two sets of coordinates. For each set of corresponding residues, it combines information pertaining to both the fraction of residues that overlap within a given RMSD window as well as those that overlap within a given distance cutoff (5.0 Å) [37].

Regions of high variability between ChxR_Eff_ and structurally characterized OmpR/PhoB effector domains exist within the transactivation loop and the disordered C-terminal extended coil. The transactivation loop is a site of variable function within this subfamily of DNA binding proteins. Not surprisingly, sequence conservation is completely absent within this region for the aligned OmpR/PhoB subfamily members (**Fig. 3**). Interactions with sigma factors (PhoB [49], [50]) or the α-subunit of RNA Polymerase (OmpR [51]) have been documented, albeit based largely upon genetic studies. Loop orientation is most similar between ChxR and OmpR, however differences do exist within this region between the two proteins. The length of the loop in ChxR_Eff_ is one residue shorter than OmpR and the residues that comprise their respective loops are quite different. Polar and charged residues primarily comprise the transactivation loop in OmpR, whereas the loop in ChxR_Eff_ is almost entirely composed of hydrophobic residues. Furthermore, the four loop residues within OmpR that are important for interaction with the α-subunit of RNA polymerase and for transcriptional activation [3] are not conserved in ChxR, suggesting an alternative site of transcriptional machinery interaction might occur.

### ChxR_Eff_ Residues that are Critical for DNA Interaction

**Figure 4:**
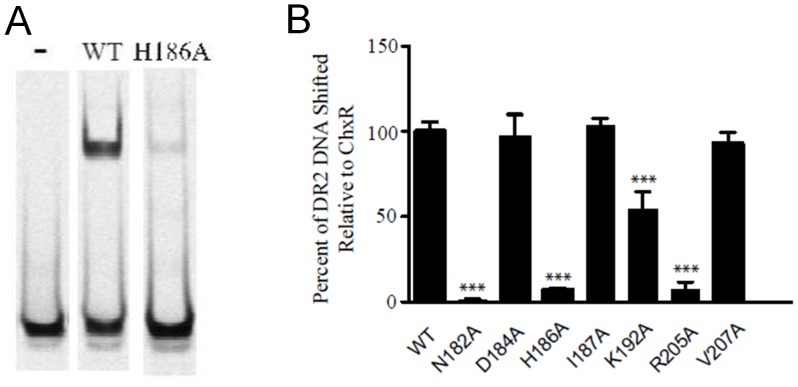
Site-directed mutagenesis identifies DNA-binding residues within ChxR. *A,* Alanine substitutions were generated in several of the residues within regions (α5–α6 and β11–β12) known to interact with DNA in other OmpR/PhoB subfamily members. Representative EMSA of IR800-labeled DNA in the absence of ChxR (-) and in the presence of 44 μM wild-type ChxR or 44 μM ChxR^H186A^. *B,* The amount of DNA shifted with each substitution was quantified. DNA binding of four substitutions (Asn182, His186, Lys192, and Arg205) was significantly (p<0.001 (***)) reduced relative to wild-type ChxR.

Residues important for direct interaction with DNA in OmpR/PhoB subfamily members are generally located within the helix-turn-helix or wing of the effector domain (**Fig. 2**) [41]. To begin identification of ChxR residues that might interact with DNA, Ala substitutions were generated within the full-length protein in surface-exposed residues of the DNA binding helix (α6) or wing (β11–β12 loop) (**Fig. 2C**). Proper folding of ChxR mutants was assessed by size exclusion chromatography and the ability of each mutant to bind DNA was evaluated using EMSAs. The amount of DNA bound by ChxR and shifted with each substitution was quantified and compared to wild-type ChxR (**Fig. 4**). Substitutions in three residues (Asn182, His186, and Lys192) within the recognition helix (α6) and a residue (Arg205) within the wing (β11–β12 loop) significantly reduced DNA interaction, while Asp184, Ile187 and Val207 substitutions appeared to bind with near wild type affinity (**Fig. 4B**). Substitution of Arg191 resulted in protein expression localized to inclusion bodies, potentially reflecting a role in structural stability of the effector domain. Importantly, residues implicated in DNA binding are not conserved amongst OmpR/PhoB subfamily members (**Fig. 3**), supporting that these may provide DNA sequence specificity for ChxR [4].

### Small-Angle X-Ray Scattering (SAXS) of ChxR Supports Conformation of Activated OmpR/PhoB Subfamily

Due to the paucity of full-length structures of OmpR/PhoB subfamily RRs in the active state, little is known structurally about receiver-effector domain interactions and effector domain orientation upon phosphorylation. While ChxR is a member of the OmpR/PhoB subfamily, it is an atypical response regulator, meaning that dimerization and function are retained in the absence of phosphorylation [15], [52], [53]. Previous biochemical characterization of ChxR demonstrated that stable homodimers, primarily through receiver domain interactions, were maintained in solution both *in vitro* and *in vivo* [17]. The results described here, in concert with previous receiver domain structural studies [13], lead us to hypothesize that ChxR exists as a constitutively active dimer in solution. Full-length ChxR has been recalcitrant to crystallization, aggregating rapidly at concentrations higher than ∼5 mg/ml (unpublished data), however, the addition of 5% (v/v) glycerol to the protein solution described within the *Materials and Methods* section resulted in conditions that give monodisperse samples. Thus, in order to test this hypothesis Small-angle X-ray Scattering (SAXS) was used to analyze the solution state of ChxR.

SAXS, with recent technical and computational advances, has become a robust and effective technique for analyzing macromolecular structures, including the generation and experimental validation of relatively high-resolution models of proteins in solution [29], [54]–[56]. This process is particularly effective when the atomic structures of individual protein components (e.g. domains) have been determined [57], which allows the generation of extensive collections of computational models reflecting many possible conformations of the full-length protein. ‘Best fit’ model(s) of the complete macromolecule can then be selected based upon the experimental SAXS data. Together, these processes (e.g. small-angle scattering and computational modeling) provide complementary information about flexibly linked domains [34], [58], [59], shape, conformation, and assembly state in solution [29], [30], [60]. Clearly, a fundamental strength of SAXS analysis is that it provides an efficient and powerful way to experimentally test models for different macromolecular assemblies and conformations in solution, as evidenced by the wealth of recent solution studies on multidomain proteins [60]–[65].

SAXS data were collected and analyzed on full-length ChxR. Concentration dependent scattering (from interparticle interference or aggregation) can be revealed by superposition of scaled scattering curves at multiple concentrations (1.0 mg/ml to 5.0 mg/ml**, Fig. S4A**). Linear dependence between Intensities, *I*(q), and concentrations indicated a systematic influence of individual scattering factors, *S*(q), up to q <0.05 Å^−1^ (**Fig. S4B**). As this q value was outside the first Shannon channel (q_min_ = 0.031 Å^−1^ for D_max_ = 100 Å; [66]), infinity dilution [29] was applied to the SAXS profile at q <0.05 Å^−1^ and merged with the higher concentration (5 mg/ml) SAXS profile at q >0.05 Å^−1^. This scattering profile was used for subsequent data analysis. The resulting Guinier plot (**Fig. S4C**) was linear, which indicated the sample was relatively free of aggregation and gave a radius of gyration of 30.8±0.3 Å. Estimated molecular mass using the Porod Volume was ∼62 kDa, which is consistent with the dimeric state of ChxR (calculated MW = ∼56 kDa). All further scattering analyses were determined from this interference free (e.g. aggregation) SAXS curve. Analysis of the Kratky and Porod-Debye plots (**Fig. S5**) reveals that ChxR may have partially unfolded or flexible regions (Porod-Debye Exponent, P = 3.8). Additional SAXS data collection parameters are available in **Table 2**.

**Figure 5:**
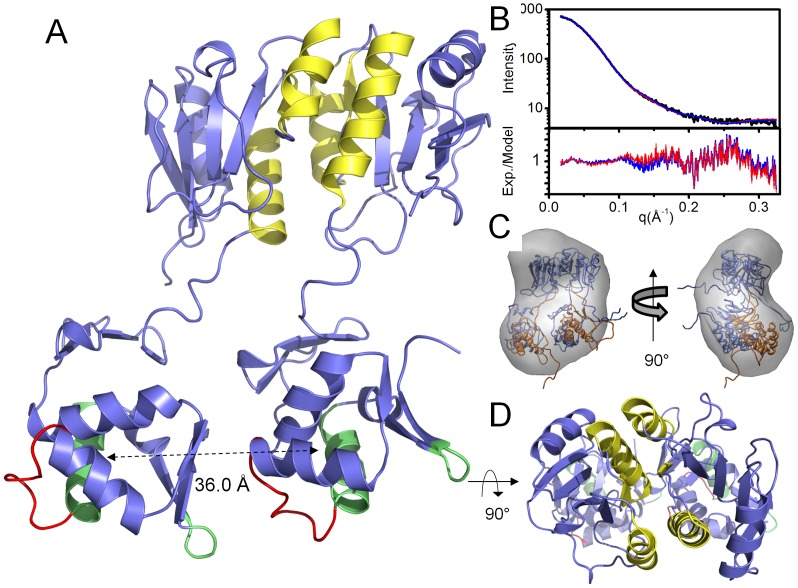
Overall arrangement of full-length ChxR dimer in solution. *A,* Cartoon ribbon format of ChxR dimer (colored blue, yellow, green and red) that had the best scattering profile fit and accounts for the entirety of the experimental scattering from inset plot. For reference, α4–β5–α5 dimer interface colored *yellow*, DNA recognition helix and wing colored *green* and transactivation loop colored *red* within each polypeptide. Effector domains are 36.0 Å apart, as measured from Ala188 Cα on each polypeptide. *B,* Experimental scattering profile (*upper graph*) of full-length ChxR dimer (black) with the single best BILBOMD-derived [33] model fit to the experimental data (χ = 3.86) (red) and MES [33] fit of the two conformers shown in panel C (χ = 3.64) (blue). Calculation of Residual Fit, Experimental Intensity divided by Model Intensity (*lower graph*). *C,* Two views of the average SAXS shape with two MES ChxR models (red fit, panel B) in ribbon format. Single best fit conformer (blue) represents ∼90% of scattering, while the 2^nd^ MES-derived conformer (orange) accounts for the remaining ∼10% of scattering. *D,* ChxR dimer rotated 90° about the horizontal plane from panel A.

**Figure 6:**
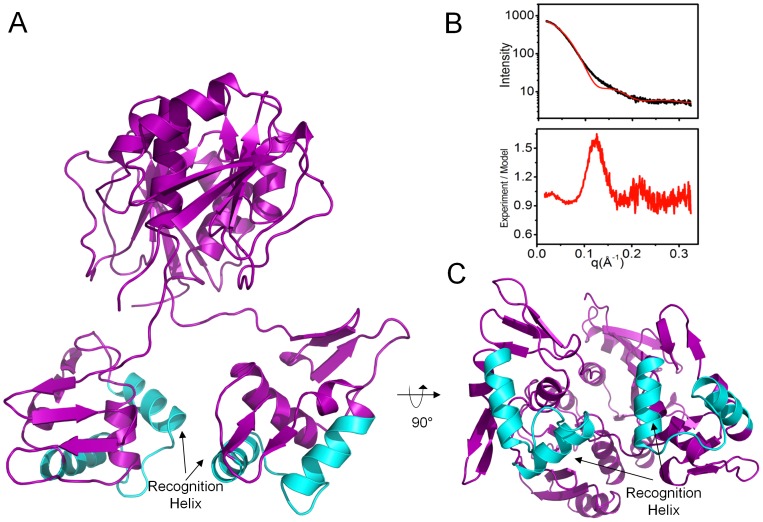
Overall arrangement of the Best-Fit Unconstrained ChxR Dimer in Solution. *A,* Cartoon ribbon format of head-to-head ChxR dimer (colored purple and cyan) that had the best scattering profile fit of the experimental scattering (*panel B*). For reference, helix-turn-helix motif colored *cyan* within each polypeptide. *B,* Experimental scattering profile (*upper graph*) of head-to-head ChxR dimer (black) with the single best BILBOMD-derived [33] *unconstrained* model fit to the experimental data (χ = 8.05) (red). Calculation of Residual Fit, Experimental Intensity divided by Model Intensity (*lower graph*). *C,* ChxR dimer rotated 90° about the horizontal plane from *panel A*.

**Figure 7:**
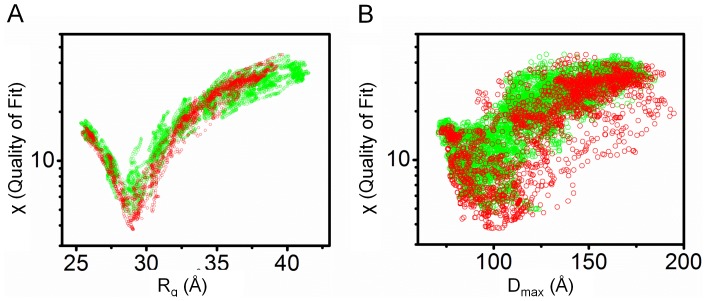
Experimental Fit for Constrained and Unconstrained Model Pools. A, Plot of χ versus R_g_ (Å) for all generated ChxR conformers. B, Plot of χ versus D_max_ (Å) for all generated ChxR conformers. In both panels, green and red circles represent *unconstrained* and *constrained* model pools, respectively.

Reconstitution of the solvated molecular envelope was carried out with a full-length model of ChxR generated by MODELLER [67] and Chimera [68], which was comprised of the previously determined [13] ChxR receiver domain homodimer (PDB ID: 3Q7R), ChxR_Eff_ mean minimized NMR solution structure described herein and models for the 24 residue N-terminal His-tag and 8 residue interdomain linker. The molecular dynamics simulation program, BILBOMD [33], facilitated the determination of ∼20,000 different conformations of full-length ChxR dimer models (∼10,000 each of *constrained* and *unconstrained* model sets; described in *Materials and Methods* section). The entire ensemble of both ChxR model sets was used to calculate theoretical SAXS profiles with FoXS [32]. In support of previous biological ChxR data indicating a “head-to-tail” effector domain orientation would be required to bind direct DNA repeats [13], [15], [17], the single best fit *constrained* conformer (**Fig. 5**) was in agreement with the experimental scattering curve (χ = 3.86). However, the single best fit *unconstrained* conformer (**Fig. 6**) produced a poor fit to the experimental scattering curve (χ = 8.05). This *unconstrained* conformer model is characterized by a “head-to-head” orientation for both effector domains, maintaining the two-fold symmetry present within the receiver domain. Additionally, the majority of this poor fit (**Fig. 6B**) occurs within the medium resolution range of the experimental SAXS profile (q = 0.1–0.2 Å^−1^), indicative of an incorrectly modeled domain conformation [29]. Analysis of the experimental scattering data with *constrained* versus *unconstrained* conformers indicated a better relative fit across all *constrained* models (**Fig. 7**). Overall, the observations from the comparative (*e.g. constrained* vs. *unconstrained*) analyses provided stronger support for the ChxR DNA binding domain being in a ‘head-to-tail’ orientation, reflective of the direct symmetry required to interact with the DNA repeats found within ChxR promoter sites [13], [15], [17].

Using the *constrained* dataset, Minimal Ensemble Search (MES) was applied to determine the level of conformational heterogeneity in ChxR and develop refined conformers that better match the experimental scattering (reviewed in [34]). Briefly, MES is a weighted genetic algorithm that generates a subset of conformers based upon multiple iterative modifications of highly representative models and best-fit selection with experimental data. Two conformers representative of the ChxR *constrained* dataset (including the single best fit conformer) were together compared to the experimental curve (**Fig. 5B, C**), achieving a slightly better fit (χ = 3.64) than the single best fit conformer alone to the experimental curve (χ = 3.86). The slightly improved χ score reflects a better fit to the experimental scattering by accounting for the *coexistence* of multiple solution conformations following MES. Importantly, the addition of more than two conformations failed to increase the quality of fit to the experimental SAXS curve, indicating that ChxR adopts a compact, dimeric conformation with a minimal degree of flexibility between each effector domain (**Fig. 5C**). As such, the entirety of the scattering profile can essentially be attributed to ChxR in a compact state with effector domains in a “head-to-tail” orientation. Moreover, the DNA binding helices within each effector domain are ∼36 Å apart, as measured from the Ala188 Cα of each chain (**Fig. 5A**). This orientation of ChxR potentially allows for each effector domain to interact within the DNA major groove of two recognition sites of the ChxR promoter, as the distance between major grooves is ∼34 Å [69].

## Discussion

### Flexibility of Interdomain Contacts in OmpR/PhoB Subfamily Members

**Figure 8:**
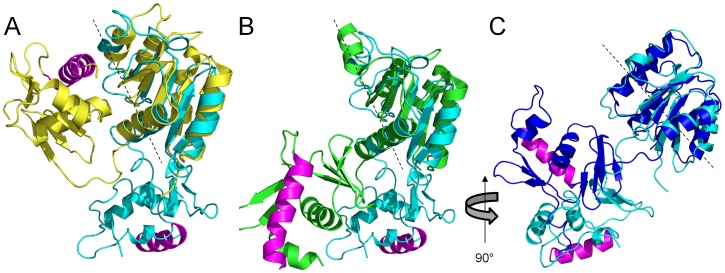
Structural comparisons of the ChxR solution state with various classes of full-length OmpR/PhoB subfamily structures. *A,* Structural superposition, through the receiver domain, of a full-length ChxR monomer (cyan) with a representative “closed” subclass full-length structure, MtrA (gold; PDB ID: 2GWR) from *M. tuberculosis. B,* Structural superposition, through the receiver domain, of a full-length ChxR monomer (cyan) with a representative “open” subclass full-length structure, DrrD (green; PDB ID: 1KGS) from *T. maritima*. ChxR is oriented as in panel A. *C,* Structural superposition, through the receiver domain, of a full-length ChxR monomer (cyan) with a representative “free” subclass full-length structure, HP1043 (blue; PDB ID: 2HQR) from *H. pylori*. ChxR is rotated 90° from panels A and B. In panels A-C dashed lines represent the α4–β5–α5 dimerization interface, while recognition helices are colored magenta.

Full-length RRs, including ChxR, have proven quite recalcitrant to structural determination, which likely is a reflection of the highly flexible interdomain interfaces formed by the receiver and effector domains. As such we propose that the available structures can be classified into three structural and functional subclasses based upon interdomain interactions and the resulting steric hindrance of the DNA recognition helix within the effector domain. The full-length structures of MtrA [6], DrrB [8] and PrrA [7] form extensive contacts between the receiver and effector domains (involving the activated dimer interface, α4–β5–α5) and thus belong to the “closed” subclass (**Fig. 8A**). Interdomain interfaces within these subclass members have been demonstrated to inhibit both *in vitro* and *in vivo* autophosphorylation rates [5]. Full-length crystal structures of RegX3 [9] and DrrD [11] have solvent accessible recognition helices as a result of limited interdomain contacts that do not primarily involve the α4–β5–α5 interface, and as such have been classified in the “open” subclass (**Fig. 8B**). Finally, we propose a new subclass of full-length RRs, termed the “free” subclass (**Fig. 8C**) whose members completely lack interdomain interactions and readily form phosphoryl-independent homodimers through the α4–β5–α5 interface. The subclass is currently comprised of HP1043 [12] and ChxR. NMR relaxation data on dimeric HP1043 supports its classification as a “free” subclass member, as residues potentially involved in interdomain interactions had an increased S^2^ (generalized order parameter) relative to the remainder of the protein [12]. In agreement with these data, NMR chemical shift perturbation experiments with varying molar ratios of ChxR_Eff_ and ChxR_Rec_ failed to detect interdomain interactions (unpublished data).

These structural subclasses are merely snapshots of an equilibrium that exists between inactive and active conformational states for prototypical OmpR/PhoB RRs [5]. The active state is stabilized by phosphorylation, which enhances DNA binding affinity, and makes dimerization through the α4–β5–α5 interface energetically favorable. Atypical RRs exist in a constitutively active, dimeric state as demonstrated by previous studies on ChxR [13], [17] and HP1043 [12], [14], [52]. Recent studies by Barbieri *et al.* demonstrated that interdomain interfaces stabilize the inactive state and inhibit phosphotransfer-mediated activation [5]. OmpR/PhoB subfamily RRs classified in the “free” subclass lack interdomain interactions and its members (ChxR and HP1043) are able to bind DNA in a phosphoryl-independent manner [10], [12], [17]. The structural studies discussed herein provide further support for the delineation of three separate OmpR/PhoB RR structural subclasses.

### DNA Interactions in OmpR/PhoB Subfamily Members

OmpR/PhoB subfamily RRs regulate a diverse collection of biological processes involving signaling, metabolism and development, among others. As such, great diversity in the target DNA sequences of these members is not unexpected. This naturally leads to unique protein-DNA interactions among subfamily members. Residues critical for DNA binding have also been identified in OmpR [43], PhoB [41], PhoP [44], KdpE [45] and HP1043 [12]. All of these residues are localized to the recognition helix (labeled α6 in **Fig. 2**) or the minor groove binding wing. Of the residue positions implicated in multiple OmpR/PhoB subfamily member mutants (*black star* in **Fig. 3**), all three predominantly involve Arginine (including Arg205 described within), the most frequent side chain involved in protein-DNA interactions [70]. This suggests these side chains are involved in non-specific DNA interactions found across various OmpR/PhoB subfamily members. Residues identified in only a single member of the OmpR/PhoBs subfamily (*blue triangles* in **Fig. 3**) predominantly involve non-conserved positions without a preference for aliphatic or charged side chains, suggesting potential roles in site-specific interactions. As expected, amino acids within the recognition helix that stabilize effector domain tertiary structure (positions 183, 187, 190 and 194; ChxR numbering) have not been implicated in DNA binding. Based up the previous observations, residues imparting protein specific-DNA base recognition are likely to be found within the few non-conserved side chains of the OmpR/PhoB recognition helix. The effect of substitutions at these specific-DNA base recognition sites in ChxR was not evaluated for their capability to bind to alternate ChxR recognition sites (e.g. DR1, 3–6; [17]). While the proposed ChxR binding motif has an overall low nucleotide conservation, three nucleotides are (TXGAXXX) are highly conserved among ChxR binding sites. Additionally, when these conserved nucleotides were mutated and naturally variant, ChxR binding was severely reduced [17]. These observations would support that the amino acids important for ChxR binding to DR2 site, and these cognate conserved nucleotides, are also important for binding to alternate sites as well. Clearly, experimental analyses will be needed to support this hypothesis.

### DNA Homology Model of ChxR Bound to Direct Repeat

**Figure 9:**
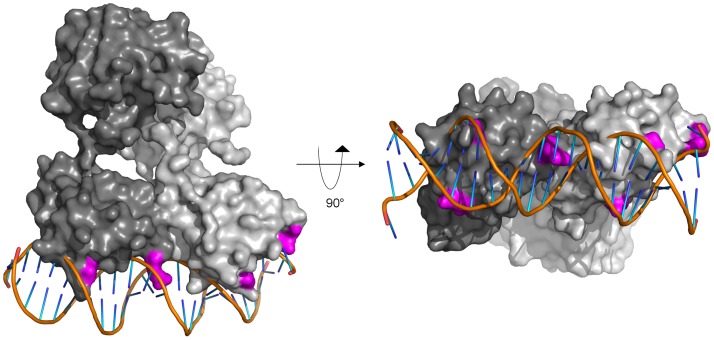
Proposed model of full-length ChxR binding to tandem DNA repeat. The solution state of full-length, dimeric ChxR (each polypeptide colored a different shade of gray, surface representation) was manually overlayed onto the cocrystal structure of the PhoB effector domain from *E. coli* bound to its cognate *pho box* (PDB ID: 1GXP). The structure is rotated 90° about the protein:DNA interface on the *right*. Surface exposed residues within ChxR that were implicated in DNA binding by site-directed mutagenesis are highlighted in magenta.

The large majority of OmpR/PhoB subfamily RRs have been found to bind direct DNA repeats, which thus requires these proteins to form functional dimers [71], [72]. Structural studies of full-length OmpR/PhoB RRs bound to their cognate DNA repeats have proven elusive, with only the PhoB effector domain in complex with the *pho* box having been reported [41]. While a full-length structure for PhoB has yet to be determined, the structures of each individual domain are available, in addition to the BeF_3_^–^activated α4–β5–α5 receiver dimer [73]. Furthermore, autophosphorylation of PhoB suggests it has a minimal interdomain interface, much like ChxR [5]. Each of these structures demonstrates strong structural similarity with the respective ChxR domain (**Fig. S6**). Of the 17 amino acid contacts within the PhoB-DNA complex, 9 are conserved within ChxR. Furthermore, the majority of the contrasting side chain interactions can be found within the recognition helix, which is anticipated given the differences in target DNA sites [17], [41]. These similarities suggested that the active state ChxR structure could be modeled onto the PhoB-DNA complex (**Fig. 9**). The four ChxR residues that were demonstrated by site directed mutagenesis to be critical for DR2 interaction are within appropriate distances to bind each direct DNA repeat. Thus, the solution structure of ChxR in a DNA-binding state provides a model for comparison within the OmpR/PhoB subfamily. However, as atypical RRs appear to lack interdomain interfaces, the primary site for regulation of prototypical RRs, further studies are needed to elucidate how these proteins are turned “on” and “off”.

The proposed model of full-length ChxR has the domains (receiver and effector) in different paired orientations. Specifically, the regulatory domains have been determined to interact with two-fold symmetry, which is strongly supported by crystallographic data for ChxR and almost all other OmpR/PhoB subfamily members. However, the DNA binding domains of ChxR appear to have tandem symmetry (head-to-tail) based upon the SAXS analysis presented herein, as well as their monomeric state and reduced DNA binding affinity in the absence of the receiver domain. This matches the orientation of the DNA sequence motif (direct repeat) recognized by ChxR [13]. The resulting orientation is not unexpected for an OmpR/PhoB subfamily member, and was recently highlighted by Bachhawat *et al.* [73]. They suggested that orientational constraint is lost between the domains of PhoB, based upon the observations of the receiver domain structure (two-fold symmetry) and DNA repeat-bound effector domain (tandem symmetry) [41]. This prediction was further strengthened by the recently reported crystal structure of PhoB in complex with RNAP and DNA [74].

Support for the ChxR-DNA model is limited by the relative structural absence of effector domains bound to DNA with only two examples of protein bound to neighboring sites. However, the overall expectation of independent domain orientation is further supported as most OmpR/PhoB subfamily response regulators bind to direct repeats of DNA sequences, which would seemingly require two similarly oriented winged helix DNA binding motifs. One of the few OmpR/PhoB subfamily members that bind to inverted repeats is the atypical response regulator HP1043 [75]. It is also among the few full-length structures from this family that have been determined and, of note, the DNA binding domains are oriented similar to the receiver domain with two-fold symmetry, which is in the best orientation for binding inverted repeats. Clearly, the absence of a full-length structures from the OmpR/PhoB subfamily bound to tandem repeat DNA limits any strong conclusions regarding the orientation of these molecules, although proposing a model that incorporates domain independent orientation seems best supported by the overall observations for OmpR/PhoB subfamily response regulators, including the structural data presented herein.

Lastly, the recent advances in *Chlamydia* genetics [76], [77] and the development of molecular tools [78]–[80] will enable studies related to the function and role of ChxR to be performed directly in *Chlamydia*. Observations described herein are essential in directing those future studies, specifically the possibility of generating dominant negative ChxR variants. Substitutions that rendered ChxR incapable of binding to DR2 still retained the ability to form homodimers. Furthermore, prior studies demonstrated that binding to both direct repeats was essential to stabilizing the protein-nucleic acid complex [17]. Thus, conditional expression [79] of DNA binding deficient ChxR could form heterodimers with wild-type ChxR and effectively disrupt the function of ChxR in *Chlamydia*. This would enable phenotypic and functional studies (e.g. transcriptome analysis) to provide a better understanding of the role of ChxR in the chlamydial developmental cycle and pathogenesis.

## Supporting Information

Figure S1 **DNA-binding analysis of ChxR_Eff_.** To determine if ChxR_Eff_ can interact with DNA in the absence of the receiver domain, EMSAs were performed with IR800-labeled DNA corresponding to the DR2 site (1 nM) from the *chxR* promoter and increasing concentrations (50 nM, 100 nM, 500 nM, 1 μM, 5 μM, or 10 μM) of recombinant ChxR_Eff_. The first lane (left) contains DNA in the absence of ChxR_Eff_.
(TIF)

Figure S2 **Superposition of 25 lowest energy conformers of ChxR_Eff_** (β-strands and α-helices are colored cyan and red, respectively) NMR solution structure.
(TIF)

Figure S3 **Structural Superposition of OmpR/PhoB Effector Domains.** Stereo view of OmpR/PhoB effector domain structures in ribbon format. Structures correspond to the following proteins/organisms: ChxR, *C. trachomatis* (gray); OmpR, *E. coli* (green); PhoB, *E. coli* (yellow); KdpE, *E. coli* (blue); PhoP, *M. tuberculosis* (orange) and HP1043, *H. pylori* (magenta).
(TIF)

Figure S4 **Experimental Scattering Profile, Guinier Plot and P(r) function of ChxR.** *A*, Experimental scattering profile of ChxR for 5 mg/ml (blue), 2.5 mg/ml (magenta), 1.25 mg/ml (green) and extrapolated curve at the infinity dilution (cyan). *B,* Intensities obtained for scaled SAXS profiles (*panel A*) at q = 0.02, 0.03 and 0.05 Å^−1^ indicate effect of the Structure factor at higher protein concentration for q <0.05 Å^−1^. To eliminate this effect we used infinity dilution for further data analysis *C,* Guinier plot with Guinier region. A linear dependence of ln(I(q)) vs. q^2^ indicates the sample is free of aggregation. Radius of gyration (R_g_) values as obtained from Guinier plot: Glmn R_g_ = 30.8±0.3 Å. *D,* Pair distribution function P(r) calculated from the SAXS curve shown in Figure 5B.
(TIF)

Figure S5 **Kratky and Porod-Debye Plot of ChxR.** *A,* Experimental SAXS curve shown as a Kratky plot indicate minimal flexibility. *B,* We performed a Porod–Debye analysis to obtain direct insights into their flexibility. In a plot of the normalized q^4^•I(q) vs. q^4^, the positive slope and obtained Porod-Debye coefficient of P = 3.8 is consistent with inter-domain flexibility (27). This observation suggests that the ChxR C-terminus remains flexible, resulting in the upward slope in the Kratky plot at high q values.
(TIF)

Figure S6 **Structural Superposition of ChxR and PhoB Receiver and Effector Domains.** *A,* Structural superposition of a full-length ChxR monomer (cyan) model from SAXS analysis with receiver (PDB ID: 1B00) and effector (PDB ID:1GXQ) domain monomers from *E. coli* PhoB (colored green and red, respectively). *B,* Structural superposition of ChxR receiver domain dimer (colored cyan, PDB ID: 3Q7R) and BeF_3_^–^activated PhoB receiver domain dimer (colored magenta, PDB ID: 1ZES). *C,* Structures from panel A and B were superimposed by Local-Global Alignment in order to access structural similarities. Table displays quantitative analysis of all superimpositions.
(TIF)
