# Supplementary figures and images for: Atypical Response Regulator ChxR from Chlamydia trachomatis Is Structurally Poised for DNA Binding
Source: PLoS One. 2014 Mar 19;9(3):e91760. doi: 10.1371/journal.pone.0091760 (PMC3960148; doi:10.1371/journal.pone.0091760)

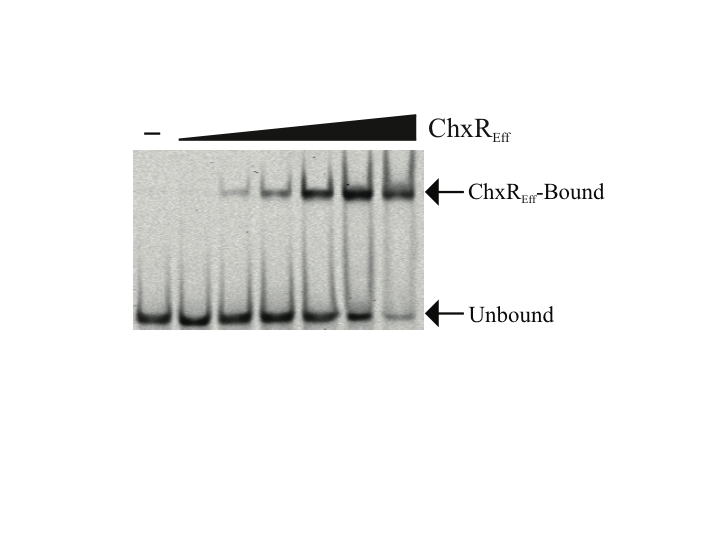

Supplement: Figure S1 — DNA-binding analysis of ChxREff. To determine if ChxREff can interact with DNA in the absence of the receiver domain, EMSAs were performed with IR800-labeled DNA corresponding to the DR2 site (1 nM) from the chxR promoter and increasing concentrations (50 nM, 100 nM, 500 nM, 1 μM, 5 μM, or 10 μM) of recombinant ChxREff. The first lane (left) contains DNA in the absence of ChxREff. (TIF) [file pone.0091760.s001.tif]

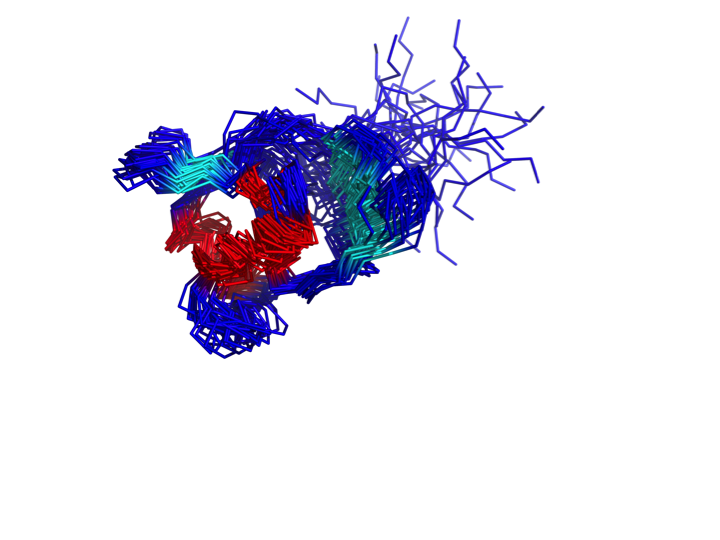

Supplement: Figure S2 — Superposition of 25 lowest energy conformers of ChxREff (β-strands and α-helices are colored cyan and red, respectively) NMR solution structure. (TIF) [file pone.0091760.s002.tif]

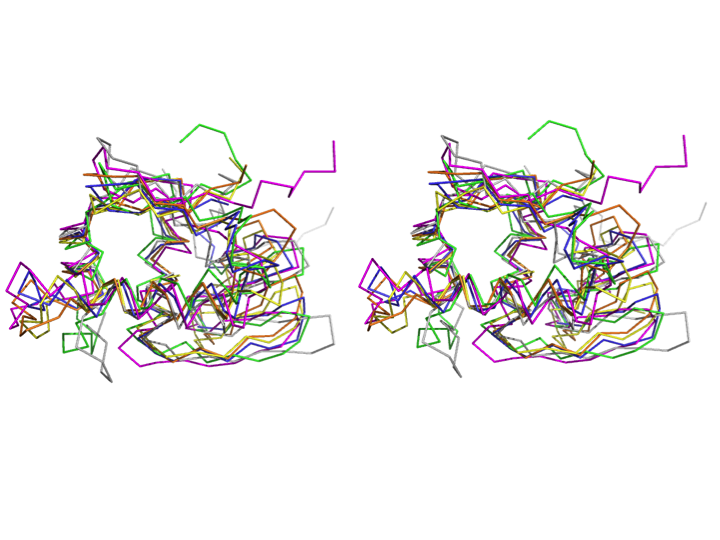

Supplement: Figure S3 — Structural Superposition of OmpR/PhoB Effector Domains. Stereo view of OmpR/PhoB effector domain structures in ribbon format. Structures correspond to the following proteins/organisms: ChxR, C. trachomatis (gray); OmpR, E. coli (green); PhoB, E. coli (yellow); KdpE, E. coli (blue); PhoP, M. tuberculosis (orange) and HP1043, H. pylori (magenta). (TIF) [file pone.0091760.s003.tif]

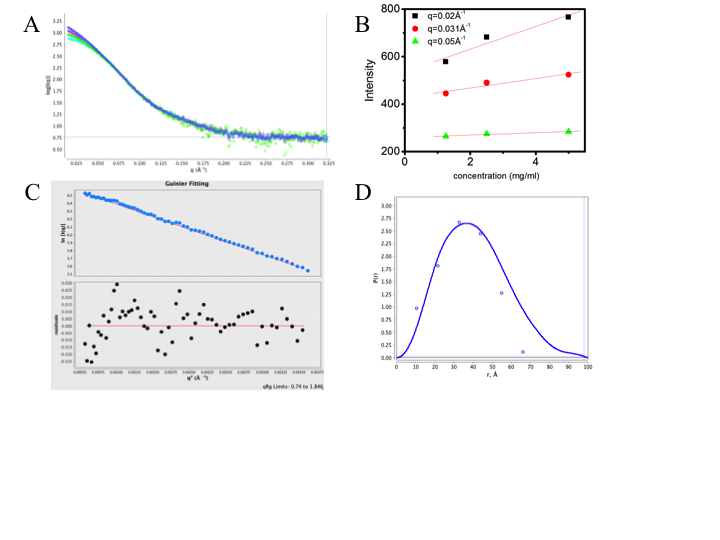

Supplement: Figure S4 — Experimental Scattering Profile, Guinier Plot and P(r) function of ChxR. A, Experimental scattering profile of ChxR for 5 mg/ml (blue), 2.5 mg/ml (magenta), 1.25 mg/ml (green) and extrapolated curve at the infinity dilution (cyan). B, Intensities obtained for scaled SAXS profiles (panel A) at q = 0.02, 0.03 and 0.05 Å−1 indicate effect of the Structure factor at higher protein concentration for q <0.05 Å−1. To eliminate this effect we used infinity dilution for further data analysis C, Guinier plot with Guinier region. A linear dependence of ln(I(q)) vs. q2 indicates the sample is free of aggregation. Radius of gyration (Rg) values as obtained from Guinier plot: Glmn Rg = 30.8±0.3 Å. D, Pair distribution function P(r) calculated from the SAXS curve shown in Figure 5B. (TIF) [file pone.0091760.s004.tif]

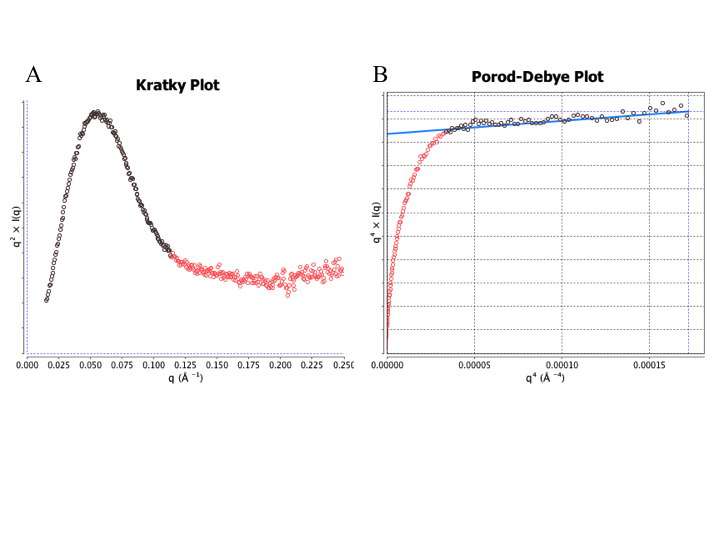

Supplement: Figure S5 — Kratky and Porod-Debye Plot of ChxR. A, Experimental SAXS curve shown as a Kratky plot indicate minimal flexibility. B, We performed a Porod–Debye analysis to obtain direct insights into their flexibility. In a plot of the normalized q4•I(q) vs. q4, the positive slope and obtained Porod-Debye coefficient of P = 3.8 is consistent with inter-domain flexibility (27). This observation suggests that the ChxR C-terminus remains flexible, resulting in the upward slope in the Kratky plot at high q values. (TIF) [file pone.0091760.s005.tif]

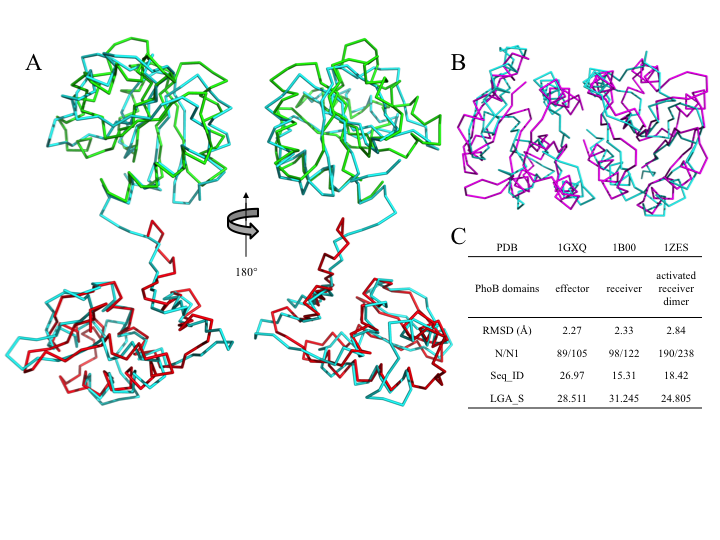

Supplement: Figure S6 — Structural Superposition of ChxR and PhoB Receiver and Effector Domains. A, Structural superposition of a full-length ChxR monomer (cyan) model from SAXS analysis with receiver (PDB ID: 1B00) and effector (PDB ID:1GXQ) domain monomers from E. coli PhoB (colored green and red, respectively). B, Structural superposition of ChxR receiver domain dimer (colored cyan, PDB ID: 3Q7R) and BeF3 –activated PhoB receiver domain dimer (colored magenta, PDB ID: 1ZES). C, Structures from panel A and B were superimposed by Local-Global Alignment in order to access structural similarities. Table displays quantitative analysis of all superimpositions. (TIF) [file pone.0091760.s006.tif]
